# Resurgence of Respiratory Syncytial Virus Infections during COVID-19 Pandemic, Tokyo, Japan

**DOI:** 10.3201/eid2711.211565

**Published:** 2021-11

**Authors:** Mugen Ujiie, Shinya Tsuzuki, Takato Nakamoto, Noriko Iwamoto

**Affiliations:** National Center for Global Health and Medicine, Tokyo, Japan

**Keywords:** respiratory syncytial virus, RSV, COVID-19, pandemic, resurgence, Tokyo, Japan, SARS-CoV-2, severe acute respiratory syndrome coronavirus 2, viruses, respiratory infections, zoonoses, coronavirus disease

## Abstract

More than a year into the coronavirus-19 pandemic, intensified infection control measures have controlled most viral respiratory infections in Tokyo, Japan. As of July 2021, however, an unusually high number of respiratory syncytial virus infections were reported in Tokyo. This resurgence may have resulted from restarting social activities for children.

The World Health Organization (WHO) declared the novel coronavirus disease (COVID-19) a global pandemic on March 11, 2020 (*1*). Since then, social activities have been restricted worldwide, and infection control measures including handwashing, mask-wearing, and keeping social distance have been strengthened. These measures reduced the prevalence of respiratory virus infections other than COVID-19, such as influenza, in 2020, and reported case numbers have sharply declined (*2*). However, the increased burden on healthcare institutions during the COVID-19 pandemic is of concern; it has and will hinder access to healthcare and the promotion of immunization programs, and the reduced number of infected or immunized persons will lead to an overall increase in susceptibility in society, leading in turn to ever-larger epidemics of infectious diseases after the resumption of social activities (*3*). We report a possible example: an unusual increase in reported cases of respiratory syncytial virus (RSV) infection in Tokyo, Japan.

We compared weekly RSV activity in the 2021 season with activity in 4 previous seasons using data from 2017–2020 from the Tokyo Metropolitan Infectious Disease Surveillance Center (Appendix Table). The center gathers the number of pediatrician-diagnosed weekly cases of RSV infection on the basis of clinical symptoms and laboratory findings from ≈260 sentinel centers, including hospitals and clinics (*4*). The most recent information is from epidemiological week 28 of 2021 (July 12–18).

No outbreaks of RSV were reported in 2020, although the previous 3 years had outbreaks in summer and autumn. However, the largest annual increase in cases since monitoring began in 2003 was reported for 2021 (Figure). The cumulative number of cases through week 28 of 2021 was 10,327, rising from a total of 570 in 2020. Whether this upward trend will continue in the latter half of 2021 is unclear as of August, but we expect the peak to be higher than in any year since 2003 and for its timing to be different. 

**Figure:**
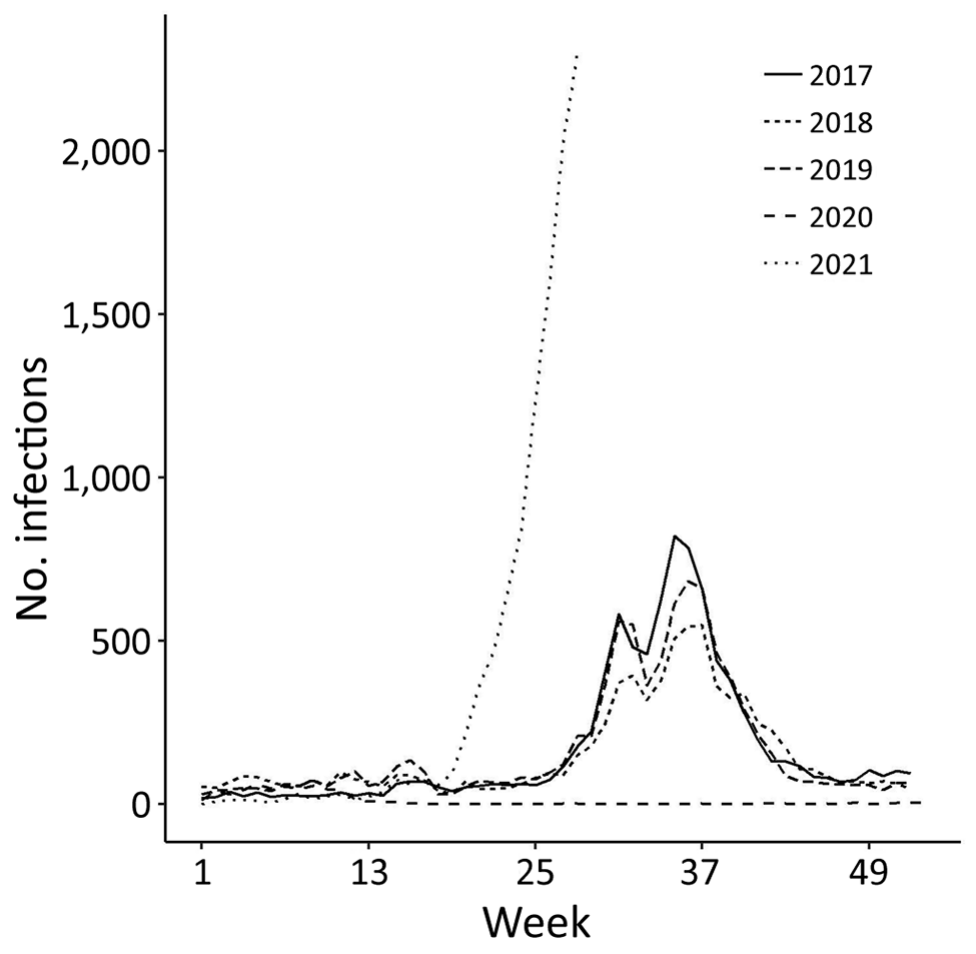
Respiratory syncytial virus infections in children, by year and epidemiological week, Tokyo, Japan, January 2017–July 2021 (as of epidemiological week 28, 2021).

Most children are infected with RSV >1 time before age 2. The statistically significant decrease in all cases reported during this epidemic compared with previous epidemics was particularly notable in children >2 years of age; by χ^2^ testing, they accounted for a significantly lower proportion of cases in 2021 than in the other years (p<0.001 for 2017, 2018, 2019, and 2020; p values corrected by the Holm method). This finding suggests that an accumulation of susceptible persons during the pandemic may have contributed to this year’s large outbreak. This trend has also been observed nationwide in Japan. In particular, the percentage of children 0–11 months of age with RSV has fallen significantly, from 32%–37% in 2018–2020 to 17% in 2021 (*5*,*6*).

The government of Japan has taken active measures to control the spread of COVID-19, including restricting children’s group activities. The government requested temporary closure of schools beginning March 2, 2020. According to the Ministry of Education, Culture, Sports, Science and Technology, 86% of schools closed for >10 weeks; 98% reopened at least partially within 14 weeks. School closures have been reported to reduce RSV epidemics, and data from Japan, which did not have an epidemic in 2020, supports this hypothesis (*7*). The importance of school life to society and children was subsequently reevaluated, however, and as a result many children now attend school. 

As of July 13, 2021, >25 million (21.9%) eligible persons >12 years of age in Japan had completed 2 doses of COVID-19 vaccination (*8*). As a consequence of vaccination, socioeconomic activity and movement have increased, and children have spent more time in schools and kindergartens than they did previously; therefore, the overall risk for infectious diseases in infants and young children is expected to increase. The increased use of schools and nurseries may also result in more aggressive diagnostic testing for alternative infections, including RSV, to rule out COVID-19, as part of the measures to prevent the spread of COVID-19 infection in patients with fever and respiratory symptoms.

The emergence of RSV epidemics after the COVID-19 pandemic, appearing in different seasons and on a different scale to previous trends, has been observed in other regions, including the Americas and Australia (*9*,*10*), and the Tokyo epidemic is therefore not unique. Nevertheless, various local factors such as nonpharmaceutical interventions, travel restrictions, viral competition, and school and nursery closures are likely to have played a role. It remains to be seen whether a similar trend will be observed with other respiratory viral infections such as influenza. New epidemics may increase the already high medical burden and cause further delays in diagnosis amid the continuing pandemic.

In summary, we report a substantial outbreak of RSV infection in Tokyo starting in spring 2021. An adequate response to this increase in patient numbers will include ongoing public infection control, restoring appropriate healthcare measures, and appropriate monitoring of the ongoing RSV epidemic.

Appendix Additional information about a resurgence of respiratory syncytial virus infections during the COVID-19 pandemic, Tokyo, Japan.
